# The maternal X chromosome affects cognition and brain ageing in female mice

**DOI:** 10.1038/s41586-024-08457-y

**Published:** 2025-01-22

**Authors:** Samira Abdulai-Saiku, Shweta Gupta, Dan Wang, Francesca Marino, Arturo J. Moreno, Yu Huang, Deepak Srivastava, Barbara Panning, Dena B. Dubal

**Affiliations:** 1 Department of Neurology, Weill Institute for Neurosciences, University of California San Francisco, San Francisco, CA USA; 2 Neurosciences Graduate Program, University of California San Francisco, San Francisco, CA USA; 3 Gladstone Institute of Cardiovascular Disease, San Francisco, CA USA; 4 Department of Biochemistry and Biophysics, University of California San Francisco, San Francisco, CA USA; 5 Bakar Aging Research Institute, University of California San Francisco, San Francisco, CA USA

**Keywords:** Cognitive ageing, Spatial memory, Hippocampus

## Abstract

Female mammalian cells have two X chromosomes, one of maternal origin and one of paternal origin. During development, one X chromosome randomly becomes inactivated^1–4^. This renders either the maternal X (X_m_) chromosome or the paternal X (X_p_) chromosome inactive, causing X mosaicism that varies between female individuals, with some showing considerable or complete skew of the X chromosome that remains active^5–7^. Parent-of-X origin can modify epigenetics through DNA methylation^8,9^ and possibly gene expression; thus, mosaicism could buffer dysregulated processes in ageing and disease. However, whether X skew or its mosaicism alters functions in female individuals is largely unknown. Here we tested whether skew towards an active X_m_ chromosome influences the brain and body—and then delineated unique features of X_m_ neurons and X_p_ neurons. An active X_m_ chromosome impaired cognition in female mice throughout the lifespan and led to worsened cognition with age. Cognitive deficits were accompanied by X_m_-mediated acceleration of biological or epigenetic ageing of the hippocampus, a key centre for learning and memory, in female mice. Several genes were imprinted on the X_m_ chromosome of hippocampal neurons, suggesting silenced cognitive loci. CRISPR-mediated activation of X_m_-imprinted genes improved cognition in ageing female mice. Thus, the X_m_ chromosome impaired cognition, accelerated brain ageing and silenced genes that contribute to cognition in ageing. Understanding how X_m_ impairs brain function could lead to an improved understanding of heterogeneity in cognitive health in female individuals and to X-chromosome-derived pathways that protect against cognitive deficits and brain ageing.

## Abstract (web summary)

The maternal X chromosome in female mice impairs cognition through the silencing of certain genes and accelerates ageing of the hippocampus.

## Main

Female mammalian cells have two X chromosomes but only one is active after embryonic development owing to random X inactivation^1–4^. Thereafter, each XX cell expresses either the maternal X (X_m_) chromosome or the paternal X (X_p_) chromosome, causing a cellular mosaicism in parent-of-X origin in the organism that varies widely among female individuals—ranging from balanced mosaicism to complete X skew^5–7^. Parent-of-X mosaicism confers both genetic and epigenetic diversity in female individuals, which could potentially buffer against dysfunction arising from processes of ageing and disease. Conversely, skew towards one parental X chromosome might increase vulnerability to effects of dysregulated processes. Thus, parent-of-X origin and its skew could influence heterogeneity of health outcomes in XX individuals. Whether X skew compared with mosaicism of the active X chromosome could contribute to organismal functions in female individuals in the absence of X mutations is currently unknown. We tested whether skew towards an active X_m_ chromosome could impair key organ functions during middle age, when age-related dysregulation begins to arise.

**Fig. 1:**
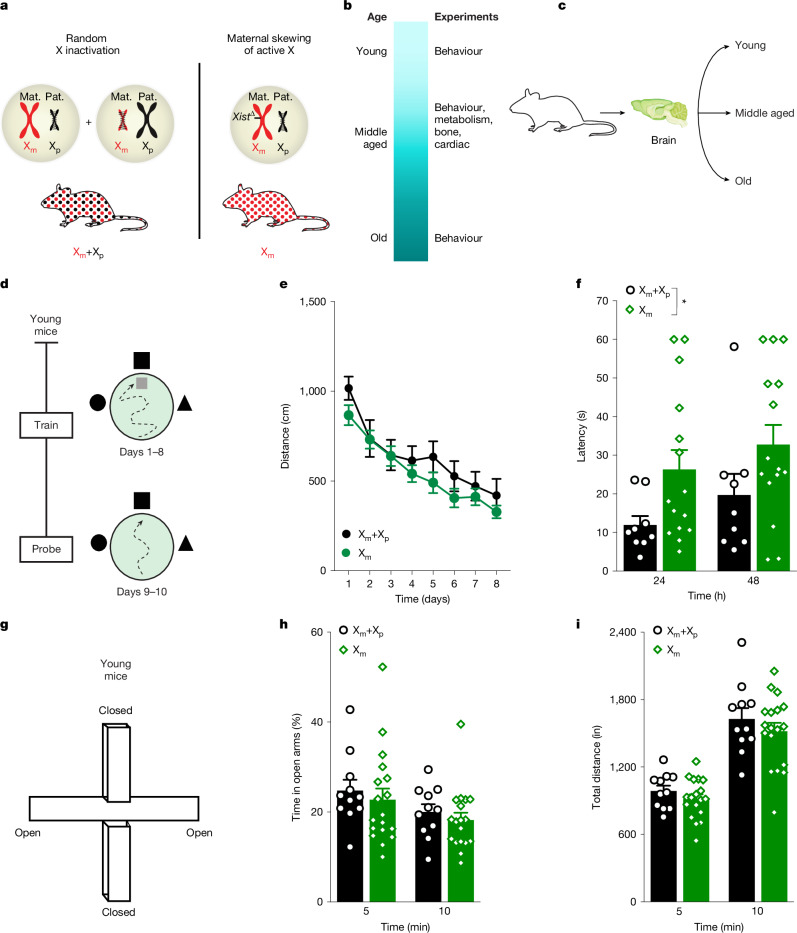
The X_m_ chromosome impairs spatial memory in young female mice. **a**, Random X-chromosome inactivation in wild-type, non-transgenic mice (X_m_+X_p_) leads to cells with either an active X_p_ or active X_m_ chromosome. In transgenic mice with X_m_ skew, all cells show only an active X_m_. Diagram of the mouse in **a** is adapted from Nadzeya Shanchuk/Shutterstock (https://www.shutterstock.com/). **b**, Experimental timeline of experiments conducted over the lifespan. **c**, Diagram of mice being tested for cognition at young, middle-aged and old life stages. Diagram of the mouse and brain in **c** are adapted from Nadzeya Shanchuk/Shutterstock and KwangSoo Kim/Shutterstock (https://www.shutterstock.com/), respectively. **d**, Schematic of the Morris water maze experiment; mice were 4–8 months old. **e**, Spatial learning in the hidden trials, measured by the distance travelled to find the platform, did not differ between the groups (two-way mixed-model analysis of variance (ANOVA)). (X_m_+X_p_ mice: *n* = 9; X_m_ mice: *n* = 15). **f**, Probe trials show that X_m_ skew impaired memory 24 h and 48 h after hidden training. Two-way ANOVA: genotype, *P = 0.0108. Bonferroni-corrected unpaired two-tailed *t*-test 24 h: *P* = 0.1092; 48 h: *P* = 0.1609 (X_m_+X_p_ mice: *n* = 9; X_m_ mice: *n* = 15). **g**, Schematic of the EPM experiment, which is used for testing anxiety-like behaviour in young mice (age: 4–8 months). **h**, Anxiety-like behaviour, measured as the percentage of time spent in the open arm, did not differ between groups at either 5 or 10 min (X_m_+X_p_ mice: *n* = 11; X_m_ mice: *n* = 18). **i**, Total distance travelled in the EPM did not differ between groups at either 5 or 10 min (X_m_+X_p_ mice: *n* = 11; X_m_ mice: *n* = 18). Each open symbol (**f**,**h**,**i**) represents an individual mouse. Data represent mean ± s.e.m. Source Data

**Extended Data Fig. 1:**
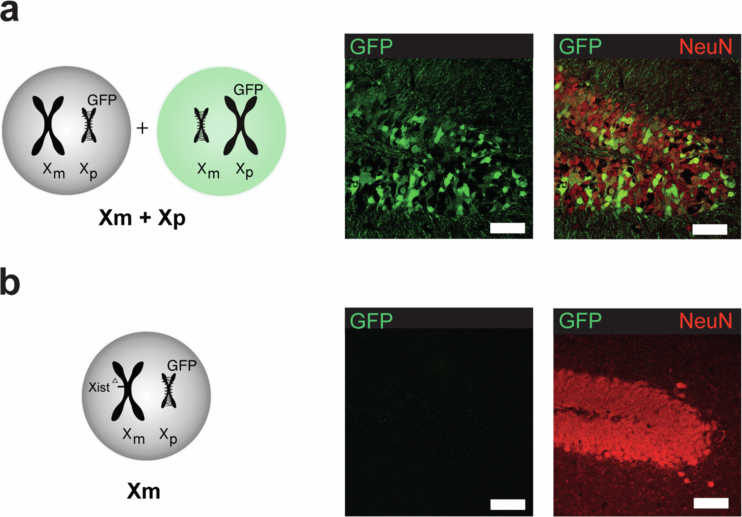
Parent-of-X mosaicism compared with paternal X silencing in maternal X skew in the hippocampus of a female mouse. **a**, Left, diagram of parent-of-X composition of cells in Xm+Xp mice. Right, Representative image of immunohistochemistry showing cells that express Xp (tagged with GFP) and all NeuN+ cells (RFP) in the dentate gyrus region of the hippocampus in a female mouse. Scale Bar = 60 mm. (n = 3 mice imaged) **b**, Left, diagram of cellular composition of cells in which the paternal X is silenced and skewed toward an active Xm (due to Xist deletion on Xm). Right, Representative image of immunohistochemistry showing maternal X skew, via paternal silencing of X (Xp, tagged with GFP) cells in the dentate gyrus region of the female hippocampus. All neurons are tagged with NeuN (red). Scale Bar = 60 μm.

We generated mice to test whether maternal X skew (called X_m_ mice here) compared with X mosaicism (called X_m_+X_p_ mice here) influences organ functions in ageing female mice (Fig. 1a). We did this by crossing mice with a targeted *Xist*-*loxP* deletion^10^ on the X_m_ chromosome, driven by a granulosa-specific Cre^11^, which enabled germline transmission. This enforces the X_m_ chromosome as the only active X chromosome. We verified mosaicism in X_m_+X_p_ mice and X_p_ silencing in X_m_ mice using immunofluorescence (Extended Data Fig. 1a,b). Because mice were on a nearly congenic C57BL/6J background (99.68–100%) (Extended Data Table 1), and *Xist* is not transcribed from the active X, differences between X_m_+X_p_ mice and X_m_ mice can be attributed to epigenetic influences.

## X_m_ and organ functions

**Extended Data Fig. 2:**
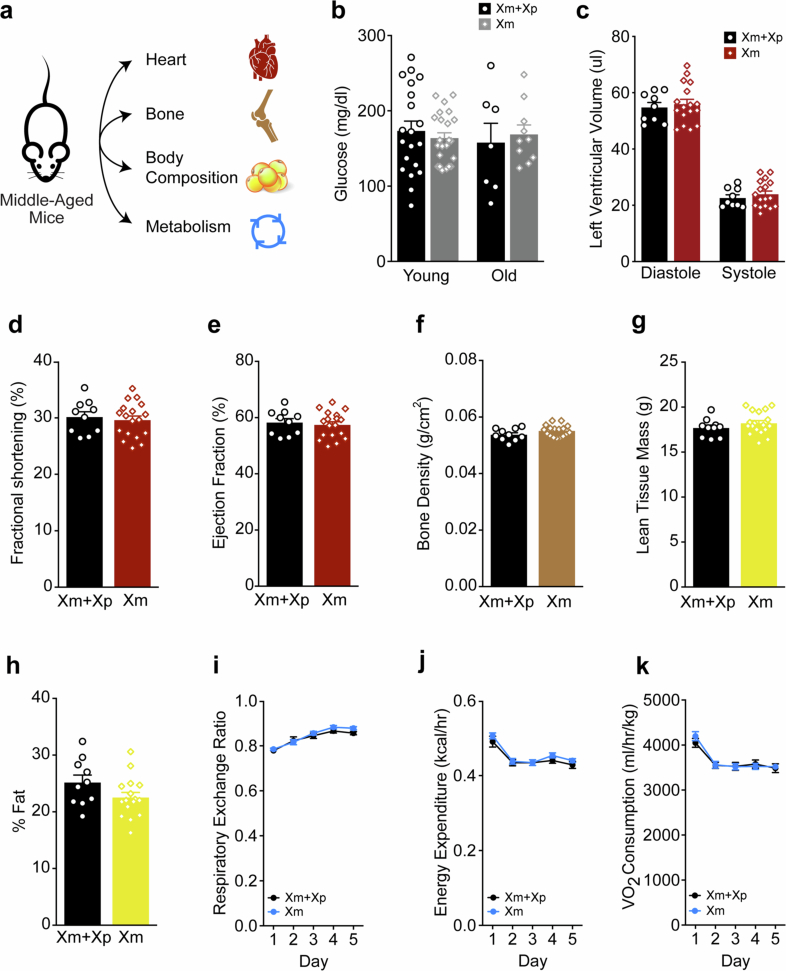
Additional measures in the body including cardiac, % fat, and V02 and VCO2 did not differ between experimental groups. **a**, Diagram of organs assessed in mice during the middle-age life stage. Diagram of the mouse, heart, bone and cells in **a** are respectively adapted from AnaitSmi/Shutterstock, Vectorfair/Shutterstock, Panda Vector/Shutterstock and Designua/Shutterstock (https://www.shutterstock.com/). **b**, Fasting blood glucose levels did not differ between Xm+Xp and Xm mice either at young (Xm+Xp mice: n = 20; Xm mice: n = 23; age = 4-8 months) or old (Xm+Xp mice: n = 7; Xm mice: n = 10; age =24-27 months) ages. **c-e**, Cardiac echo was used to measure systole and diastole, fractional shortening, and ejection fraction (age = 16-19 months). **c**, Left ventricular volume measurements in diastole and systole did not differ between Xm+Xp and Xm-only, middle-aged mice (Xm+Xp diastole: n = 9; Xm diastole: n = 17; Xm+Xp systole: n = 9; Xm systole: n = 17). **d**, Fractional shortening measurements did not differ between Xm+Xp and Xm-only, middle-aged mice (Xm+Xp mice: n = 10; Xm mice: n = 18). **e**, Ejection fraction did not differ between Xm+Xp and Xm middle-aged mice (Xm+Xp mice: n = 10; Xm mice: n = 18). f-h, DEXA-scan of the body (age = 14-17 months) to measure bone density, and tissue mass. **f**, Bone density did not differ between Xm+Xp and Xm, middle-aged mice (Xm+Xp mice: n = 10; Xm mice: n = 17). **g**, Lean tissue mass did not differ between Xm+Xp and Xm, middle-aged mice (Xm+Xp mice: n = 10; Xm mice: n = 18). **h**, Body fat percentage did not differ between Xm+Xp and Xm mice (Xm+Xp mice: n = 10; Xm mice: n = 16). **i-k**, CLAMS metabolic cages (age = 14-17 months) were used to measure metabolic parameters including respiratory exchange ratio (RER) and energy expenditure. **i**, Respiratory exchange ratio (RER) did not differ between Xm+Xp and Xm, middle-aged mice (Xm+Xp mice: n = 9-10; Xm mice: n = 17-18). **j**, Energy expenditure did not differ between Xm+Xp and Xm, middle-aged mice (Xm+Xp mice: n = 10; Xm mice: n = 17-18). **k**, VO2 consumption did not differ between the groups (Xm+Xp mice: n = 10; Xm mice: n = 17-18). Two-way mixed model ANOVA: genotype *P* > 0.05. Data represent means ± SEM. Source Data

To assess overall health, we raised littermate X_m_+X_p_ and X_m_ mice to old age and characterized measures across organ systems (Extended Data Fig. 2a). No differences in fasting blood glucose levels were detected in young mice (4–8 months) or old mice (24–27 months) (Extended Data Fig. 2b). We measured cardiac function, bone density, body composition and energy metabolism (Fig. 1b) during middle age, a life stage vulnerable to ageing-induced dysfunctions^12–16^. Echocardiography showed that the left ventricular volume, fractional shortening and ejection fraction of the heart (Extended Data Fig. 2c–e) were similar between the groups. Likewise, body composition, including bone densities, lean tissue mass and percentage fat measures (Extended Data Fig. 2f–h), did not differ. The respiratory exchange ratio, energy expenditure and oxygen consumption were similar between groups (Extended Data Fig. 2i–k). Thus, expression of only the X_m_ chromosome did not alter measured heart, bone and metabolic functions in middle-aged female mice.

## X_m_ impairs cognition

The X chromosome is enriched for genes involved in neural function^17^ and disruption of X-linked genes often causes intellectual impairments^17^. However, whether X_m_ chromosome skew, in the absence of X mutations, could influence cognition in female individuals is unknown. Thus, we next assessed behavioural and cognitive measures in X_m_ and X_m_+X_p_ mice across the lifespan, starting with young mice (Fig. 1c).

**Extended Data Fig. 3:**
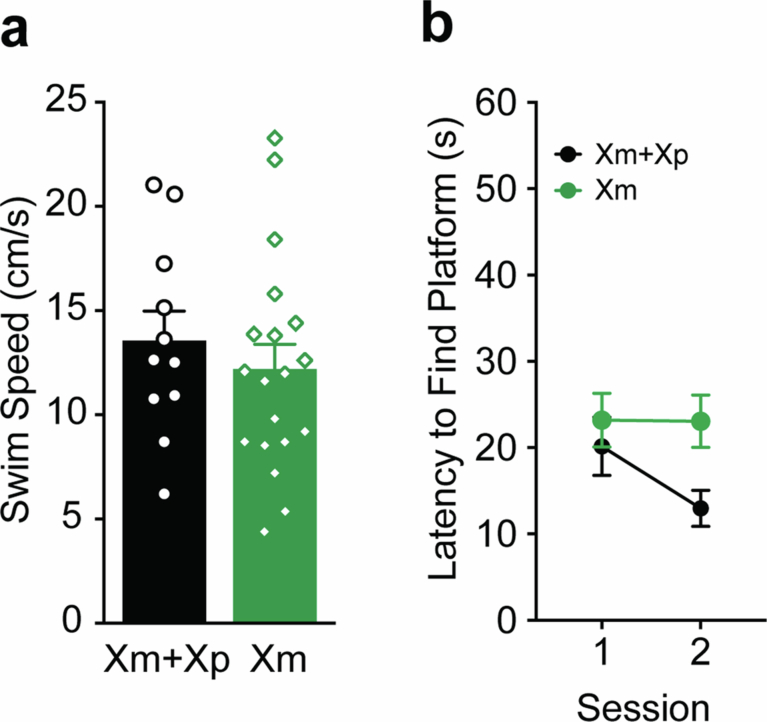
Swim speed and latency to find visible platform did not differ between groups. **a**, Swim speed did not differ between the experimental groups, measured during the visible platform trials (Xm+Xp mice: n = 11; Xm mice: n = 19; age = 4-8 months). Unpaired two-tailed t-test, *P* > 0.05. **b**, Latency to find a visible platform did not differ between the experimental groups. (Xm+Xp mice: n = 9; Xm mice: n = 15); age = 4-8 months), Two-way mixed model ANOVA: genotype *P* > 0.05. Data represent means ± SEM. Source Data

In Morris water maze experiments (Fig. 1d), which measure spatial learning and memory, both young X_m_ and X_m_+X_p_ mice (4–8 months) showed comparable spatial learning in trials with a hidden platform (Fig. 1e). By contrast, in probe trials, which measure the ability of mice to remember the platform location, X_m_ mice had impaired memory (Fig. 1f); latency to the platform location was measured since it showed a dynamic assay range for young mice. Swimming speeds and the ability to find a visible platform in the Morris water maze did not differ between the groups of young mice (Extended Data Fig. 3). Furthermore, time spent in the open arms of the elevated plus maze (EPM), which measures anxiety-like behaviour (Fig. 1g,h), along with distance travelled in the EPM (Fig. 1i), were also similar between the groups of young mice, indicating that the impairments are specifically associated with spatial memory.

**Fig. 2:**
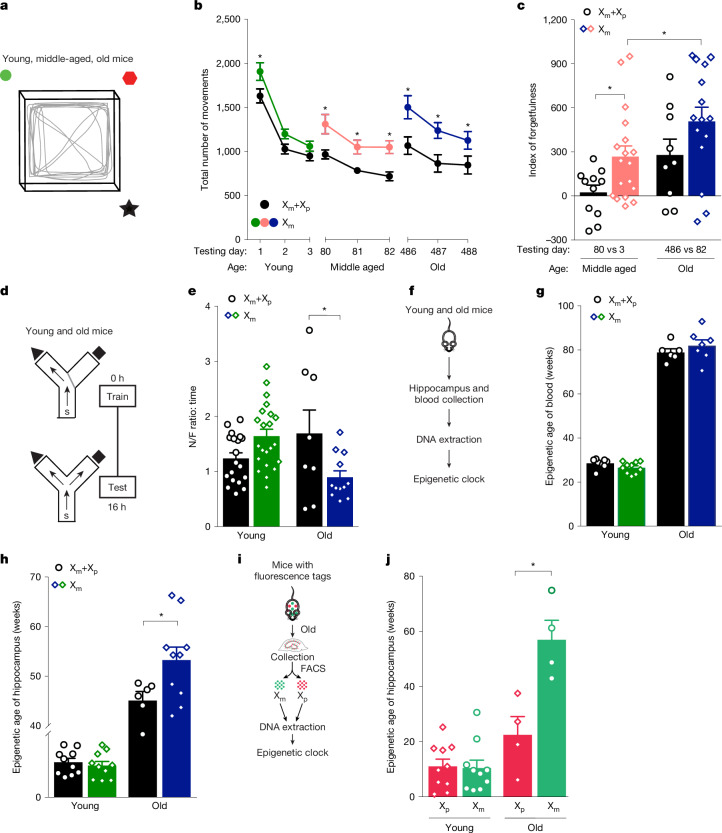
The X_m_ chromosome impairs cognition across the lifespan and accelerates epigenetic brain ageing in female mice. **a**, Diagram of the open-field apparatus to test context-dependent spatial learning and memory^18^. Mice were tested for 10 min on three consecutive days across the lifespan during young (age: 4–8 months), middle-aged (age: 9–11 months) and old (age: 20–24 months) life stages in the same cohort; day 1 is the first day of open-field testing in young mice. **b**, X_m_ skew increasingly impaired spatial habituation and dishabituation across the lifespan. *Significant or trending to significant (unpaired *t*-test). Two-way mixed-model ANOVA: time, *P* ≤ 0.0001; genotype, *P* = 0.0056 (X_m_+X_p_ young mice: *n* = 10; X_m_+X_p_ middle-aged mice: *n* = 10; X_m_+X_p_ old mice: *n* = 9; X_m_ young mice: *n* = 19; X_m_ middle-aged mice: *n* = 18–19; X_m_ old mice: *n* = 14–15). **c**, X_m_ skew accelerated age-dependent forgetfulness by middle age (day 80 versus day 3 (80 vs 3)) (unpaired two-tailed *t*-test, *P = 0.0231; X_m_+X_p_ middle-aged mice: *n* = 11; X_m_ middle-aged mice: *n* = 18). In the old mice (day 486 versus day 82 (486 vs 82)), X_m_ skew further worsened forgetfulness (unpaired two-tailed *t*-test, *P = 0.0522; X_m_+X_p_ old mice: *n* = 9; X_m_ old mice: *n* = 15). **d**, Diagram of the two-trial large Y maze for testing spatial and working memory in young mice (age: 2–5 months) and old mice (age: 20–24 months). **e**, In old mice, but not young mice, X_m_ skew impaired spatial and working memory, measured by ratio of the time spent in the novel or familiar arm. Two-way ANOVA: age by genotype interaction; *P* = 0.0011. Bonferroni-corrected unpaired two-tailed *t*-test for old mice, *P = 0.0473 (X_m_+X_p_ young mice: *n* = 19; X_m_+X_p_ old mice: *n* = 8; X_m_ young mice: *n* = 22; X_m_ old mice; *n* = 12). **f**, Diagram of experimental process for assaying epigenetic age in young mice (age: 6 months) and old mice (age: 25 months). **g**, Epigenetic age of blood did not differ between young mice or old mice (X_m_+X_p_ young mice: *n* = 8; X_m_+X_p_ old mice: *n* = 6; X_m_ young mice: *n* = 10; X_m_ old mice: *n* = 7). **h**, X_m_ increased epigenetic age of the hippocampus in the old mice. *P = 0.0443 (unpaired two-tailed *t*-test; X_m_+X_p_ old hippocampi: *n* = 6; X_m_ old hippocampi: *n* = 10), but not in young mice (X_m_+X_p_ young hippocampi: *n* = 10; X_m_ young hippocampi: *n* = 10). **i**, The experimental process for assaying epigenetic DNA age in young mice (age: 6 months) and old mice (age: 25 months). Diagram of mouse in **i** is adapted from AnaitSmi/Shutterstock (https://www.shutterstock.com/). **j**, In old hippocampal and cortical neurons, X_m_ increased epigenetic age compared with X_p_. *P = 0.0068 (paired two-tailed *t*-test; X_p_ old: *n* = 4 mice; X_m_ old: *n* = 4 mice). Epigenetic age was similar between young neurons expressing X_m_ and X_p_ (X_p_ young: *n* = 10 mice; X_m_ young: *n* = 10 mice). Each open symbol (**c**,**e**,**g**,**h**,**j**) represents an individual mouse. Data represent mean ± s.e.m. Source Data

We next assessed whether X_m_ influences spatial memory using a different test over the lifespan. Young, middle-aged and old X_m_+X_p_ and X_m_ mice were assessed for spatial memory using repeated testing in an open field^18^ (Fig. 2a). During the young life stage, X_m_ increased activity at baseline, followed by habituation, in the same spatial context, to activity levels similar to those of mosaic X_m_+X_p_ controls (Fig. 2b). During middle age, X_m_ increased forgetfulness of the spatial context compared with mosaic X_m_+X_p_ controls (Fig. 2b,c). During old age, X_m_ further worsened forgetfulness (Fig. 2b,c).

X_m_ increasingly worsened memory with age; thus, we next tested young and old mice of each group in the two-trial Y maze (Fig. 2d), a task sensitive to deficits in working and spatial memory in ageing^19,20^. During the young life stage, measures did not differ between X_m_ and X_m_+X_p_ mice (Fig. 2e). During old age, X_m_ decreased the ratio of time mice spent in the novel compared with familiar arm, indicating worsened memory compared with mosaic X_m_+X_p_ controls (Fig. 2e). Thus, X_m_ impaired working and spatial memory in old female mice.

## X_m_ accelerates brain ageing

**Extended Data Fig. 4:**
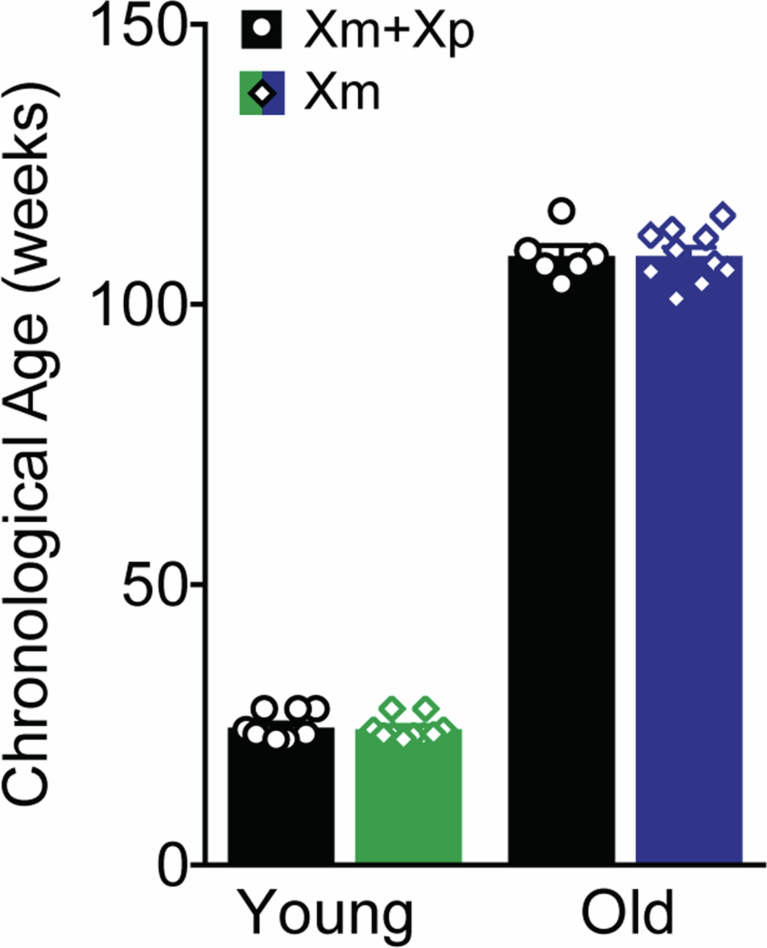
Chronological age of young mice or old mice did not differ by experimental group. Young and old Xm+Xp and Xm mice had similar chronological ages (Xm+Xp young mice: n = 10; Xm+Xp old mice: n = 6; Xm young mice: n = 10; Xm old mice: n = 10; age: young mice=6 months; old mice = 25 months. Data represent means ± SEM. Source Data

Since the X_m_ chromosome consistently worsened cognitive dysfunction with age, we wondered whether it accelerates biological ageing of the hippocampus. Ageing induces epigenetic alterations that are robust indicators of biological age^21^ (known as the ‘epigenetic clock’) measured as predictable DNA methylation patterns^22,23^. Acceleration of the epigenetic clock in one experimental group compared with another indicates increased biological ageing. To assess the relative DNA methylation of X_m_ compared with X_m_+X_p_, we analysed methylation profiles of approximately 2,045 specific age-associated DNA loci in the blood and hippocampi of young and old mice in each experimental group (Fig. 2f). Chronological ages between the experimental groups did not differ (Extended Data Fig. 4). Blood from X_m_ and X_m_+X_p_ young and old mice did not differ in biological age (Fig. 2g). In contrast to blood, biological ages in the hippocampus differed; X_m_ accelerated the epigenetic clock compared with mosaic X_m_+X_p_ controls (Fig. 2h), causing X_m_ hippocampi to be biologically older (Fig. 2h) in old mice, but not in young mice.

**Extended Data Fig. 5:**
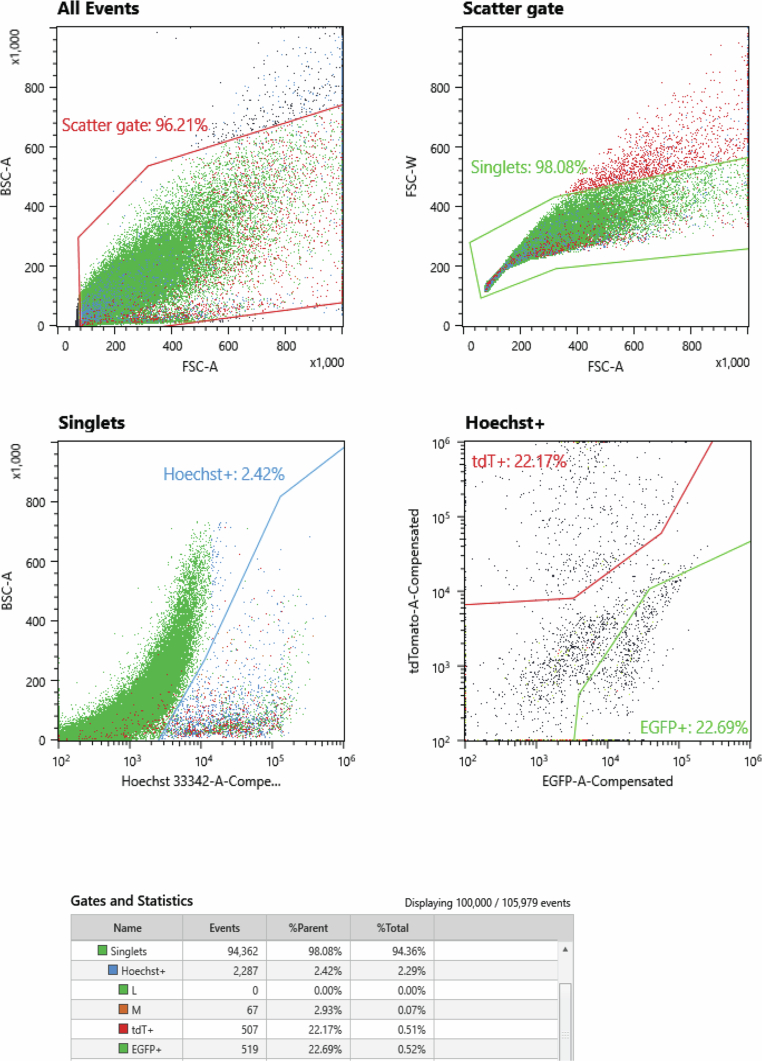
Representative FACS plots illustrating the gating strategy used to obtain GFP+ and tdT+ hippocampal neurons. Samples were first sorted by FSC and SSC to obtain single cells. Hoechst staining was then used to subset viable cells from the rest of the population. Hoechst+ cells were then separated into a tdT+ cell population and a GFP+ cell population.

To extend our findings of X_m_-mediated accelerated ageing of the hippocampus, we further examined the epigenetic clock using another transgenic mouse model that enabled separation of X_m_ from X_p_ neurons in the same brain (Fig. 2i). Using Cre-driven neuronal fluorescence through nuclear genetic reporters of the X chromosome, this model enabled neuron-specific identification of X_m_ cells (labelled with GFP) and X_p_ cells (labelled with tdTomato)^24^. Through fluorescence-activated cell sorting (FACS) (Extended Data Fig. 5), we separated X_m_ from X_p_ neurons from young and ageing female XX hippocampi that underwent random X-chromosome inactivation and conducted epigenetic clock analysis. We again found that X_m_ accelerated biological ageing in old, but not young, neurons, even when compared with neighbouring neurons expressing X_p_ (Fig. 2j). Together, these findings suggest that X_m_ accelerates biological ageing in the hippocampus, a key cognitive region targeted by ageing.

Among key cardiac, bone, metabolic and brain functions, the X_m_ chromosome selectively impaired brain function. Several lines of evidence support disproportionate influence of the X chromosome on the brain. Disruptions in X gene expression, through X-linked disorders, often cause intellectual disabilities^17,25^. Furthermore, in the brain, more genes are expressed from the X chromosome than from any other single autosome^26^. Together, these examples imply that the brain, compared with other organs, may be more sensitive to variations in X-chromosome expression.

Our experiments used two genetic models to analyse the X_m_ chromosome. In our studies of X_m_ compared with X_p_ chromosome from the same brain, the X_m_ and X_p_ chromosomes were genetically identical; thus, any differences would be attributed to epigenetic changes. Similarly, in our studies of X_m_ compared with X_m_+X_p_ mice, the X_m_ and X_p_ chromosomes were nearly genetically identical (Extended Data Table 1). Thus, X_m_ chromosome skew in this model would probably cause epigenetic differences that influence gene expression. Among these epigenetic differences, effects of the *Xist* deletion on the active X, which enforced X_m_ skew, cannot be ruled out, although it is normally silenced on the active X.

Notably, studies of female individuals with Turner’s syndrome that have only an X_m_ chromosome compared with an X_p_ chromosome show greater cognitive deficits^27^. These data in humans suggest that genes influencing cognition are imprinted or silenced on the X_m_ chromosome; however, whether X_m_ undergoes imprinting or gene silencing remained unknown.

## Epigenetic silencing by X_m_

**Fig. 3:**
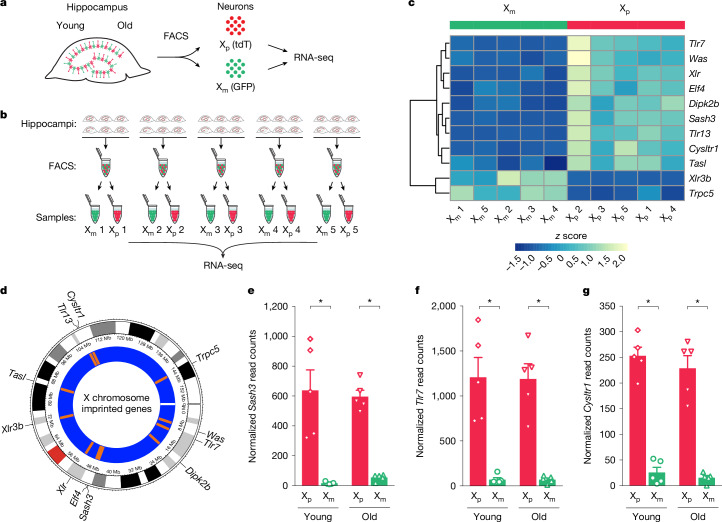
The identification of imprinted X genes on the X_m_ and X_p_ chromosomes of female hippocampal neurons. **a**, Neurons labelled with synapsin 1 driven by Cre were FACS-sorted into X_p_ (tdTomato (tdT), red) or X_m_ (GFP, green) from the same hippocampus for RNA-seq. **b**, Hippocampi of adult female mice were sorted into neurons expressing X_m_ and X_p_ (age: 3–4 months; *n* = 5 samples per group; each sample contained cells from six pooled hippocampi); these were used for RNA isolation for RNA-seq and for RT–qPCR validation. **c**, Heat map of gene expression for the top 11 imprinted genes on the X chromosome. **d**, Circos plot showing the topographical distribution of imprinted genes on the X chromosome. **e**–**g**, RNA-seq expression graphs for the top three most robustly X_m_-imprinted genes, *Sash3* (**e**), *Tlr7* (**f**) and *Cysltr1* (**g**), in neuronal samples from young and old mice (Benjamini and Hochberg-corrected Wald test, DESeq2 analysis: *Sash3* young *P = 4.0147 × 10^−32^, old *P = 3.4133 × 10^−33^; *Tlr7* young *P = 2.3316 × 10^–19^, old *P = 2.5803 × 10^–19^; *Cysltr1* young *P = 6.0631 × 10^−8^, old *P = 2.7939 × 10^−18^) (X_p_ young: *n* = 5; X_p_ old: *n* = 5; X_m_ young: *n* = 5; X_m_ old: *n* = 5). Each open symbol (**e**,**f**,**g**) represents an individual mouse. Data represent mean ± s.e.m. Source Data

We next investigated whether the X_m_ chromosome undergoes gene silencing, an epigenetic parent-of-origin effect. To achieve high resolution in this study, we applied RNA sequencing (RNA-seq) to transgenic female mice with neuron-specific labelling of the parent-of-X with X_m_ (labelled with GFP) and X_p_ (labelled with tdTomato) cells^24^. Using cell sorting, we separated X_m_ from X_p_ neurons, both genetically identical, from young and old female XX hippocampi that underwent random X-chromosome inactivation and conducted bioinformatic analyses (Fig. 3a,b). We detected 848 of approximately 1,500 known X-chromosome genes and applied established criteria to detect imprinting^28,29^.

**Extended Data Fig. 6:**
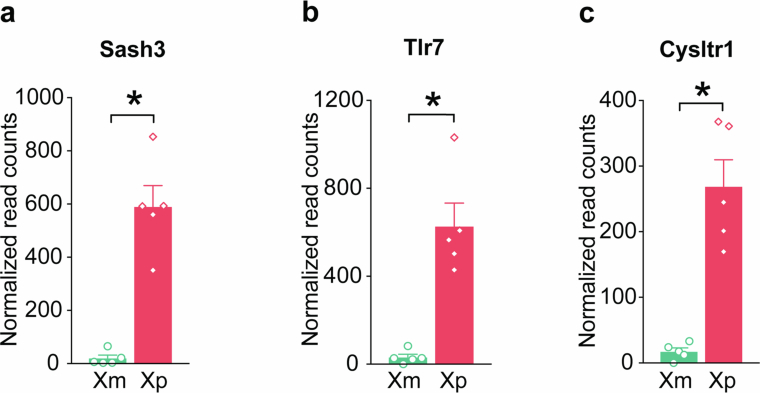
RNA-seq data replication in young mice. **a-c**, RNA-seq expression graphs for top 3 imprinted genes. **a**, Cells with active maternal X (Xm) have significantly decreased Sash3 expression compared to cells with active paternal X (Xp). Benjamini and Hochberg corrected Wald test, DESeq2 analysis: Sash3, *P = 6.20 × 10^-08 (Xp: n = 5; Xm: n = 5). **b**, Cells with active maternal X (Xm) have significantly decreased Tlr7 expression compared to cells with active maternal X (Xm). Benjamini and Hochberg corrected Wald test, DESeq2 analysis: Tlr7 *P = 1.92 × 10^-05 (Xp: n = 5; Xm: n = 5). **c**, Cells with active maternal X (Xm) have significantly decreased Tlr7 expression compared to cells with active maternal X (Xm). Benjamini and Hochberg corrected Wald test, DESeq2 analysis: Cysltr1 *P = 1.52 × 10^-10 (Xp: n = 5; Xm: n = 5). Data represent means ± SEM. Source Data

**Extended Data Fig. 7:**
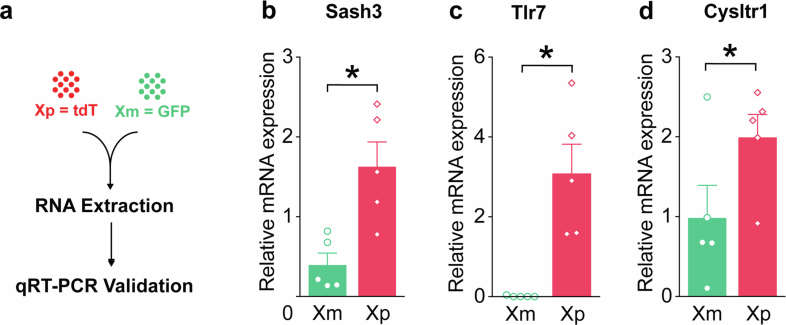
qRT-PCR validation for RNA-seq data. **a**, Diagram of qRT-PCR experimental workflow for validation of RNAseq results. RNA samples were derived from samples outlined in Fig. 3b (mice, age = 3-4 months; n = 5 samples per each group; each sample contained cells from 6 pooled hippocampi). **b-d**, qPCR validation for the top 3 imprinted genes including (b) Sash3 (*P* = 0.0230), (c) Tlr7 (*P* = 0.0068), (d) Cysltr1 (*P* = 0.0381) (Paired one-tailed t-tests). Data represent means ± SEM. Source Data

The X_m_ chromosome showed silencing or imprinting of nine genes as shown in the heat map (Fig. 3c), including *Sash3*, *Tlr7* and *Cysltr1*, the most robustly silenced genes. This was observed in hippocampi from both young and old mice. Genes were distributed throughout the X chromosome (Fig. 3d) and nearly undetectable from the X_m_ chromosome, with very high expression from the X_p_ (Fig. 3e–g). Furthermore, the X_p_ chromosome showed silencing of two genes, *Xlr3b* and *Trpc5* (Fig. 3c). *Xlr3b*, as previously identified^30,31^, showed nearly undetectable expression from the X_p_ chromosome and very high expression on the X_m_ chromosome. RNA-seq findings of X_m_ imprinting were replicated in an independent cohort of young mice (Extended Data Fig. 6a–c). Furthermore, we validated RNA-seq data in young hippocampi by real-time quantitative PCR (RT–qPCR) of *Sash3*, *Tlr7* and *Cysltr1* mRNA in young mice (Extended Data Fig. 7a) and found similar expression patterns (Extended Data Fig. 7b–d).

## CRISPR activation of X_m_-silenced genes

We hypothesized that selective X_m_ imprinting (or silencing) of genes, particularly in the network cognitive hub within the dentate gyrus of the hippocampus^32–35^, could contribute to impairment of neuronal functions underlying cognitive ageing. To test this, we determined whether gain of function (by upregulation) of select X_m_-imprinted genes could improve cognition itself, the most valuable and central manifestation of brain function that declines with age.

**Fig. 4:**
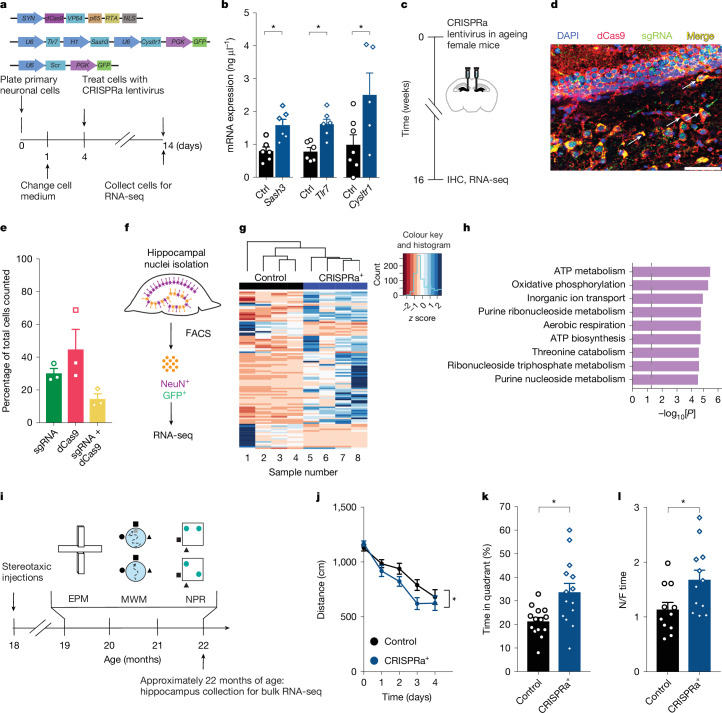
CRISPRa of maternally silenced X genes *Sash3*, *Tlr7* and *Cysltr1* improves cognition in old female mice. **a**, CRISPRa^+^ lentiviral plasmids (dCas9 + sgRNA constructs), experimental model and timeline of in vitro validation of CRISPRa^+^ cells. Scr, scrambled sgRNA. **b**, Validation using primary neuronal cultures showed CRISPRa lentivirus mediates increased mRNA expression of *Sash3* (*P = 0.0049, *n* = 6 wells per group), *Tlr7* (*P = 0.0019, *n* = 6 wells per group) and *Cysltr1* (*P = 0.0429, *n* = 7 wells control, *n* = 5 wells treatment). Unpaired two-tailed *t*-tests. **c**, CRISPRa lentivirus (dCas9 + sgRNAs) was stereotaxically injected into the hippocampus of female C57BL/6J mice (*n* = 14 mice per group, age: 18 months). Hippocampi were collected for immunohistochemistry (IHC) and RNA-seq. **d**, Representative dentate gyrus image following stereotaxic injection of dCas9 (red) and sgRNA (green) lentivirus. Scale bar, 50 µm. DAPI (blue), nuclei. White arrows indicate co-labelling of dCas9 and sgRNA. *n* = 3 mice imaged. **e**, Percentage of dentate gyrus cells expressing sgRNA (green), dCas9 (red) and yellow (co-localization of sgRNA and dCas9) (*n* = 3 mice). **f**, Transfected hippocampi were FACS-sorted to obtain GFP^+^ (sgRNA) neurons (NeuN^+^) in control and CRISPRa^+^ groups for RNA-seq. **g**, Heat map of top 100 DEGs following RNA-seq of control compared with CRISPRa^+^ hippocampi (age: 22 months; *n* = 4 samples per group). **h**, Gene ontology analysis of DEGs comparing control and CRISPRa^+^ hippocampal samples (Fisher’s exact test, PANTHER GO analysis). **i**, Experimental model and timeline of behavioural testing in old female mice. MWM, Morris water maze; NPR, novel place recognition. **j**, Spatial learning in the hidden trials, measured as the distance travelled to find the platform, shows that CRISPRa^+^ overexpression of *Sash3*, *Tlr7* and *Cysltr1* improved learning compared with controls (control mice: *n* = 14–15; CRISPRa^+^ mice: *n* = 13–14 mice; age: 20 months). Two-way mixed-model ANOVA: treatment, *P = 0.0154. **k**, Probe trials show that CRISPRa^+^ improved memory after hidden training (*n* = 14 mice per group, age: 20 months). Unpaired two-tailed *t*-test, *P = 0.0054. **l**, CRISPRa^+^ in mice improved spatial memory measured as the increased time spent with the object at the novel position, compared with control mice (control mice: *n* = 11; CRISPRa^+^ mice: *n* = 12). Unpaired two-tailed *t*-test, *P = 0.0233. Data represent mean ± s.e.m. Source Data

We applied CRISPR activation (CRISPRa) technology in neurons to simultaneously upregulate expression of *Sash3*, *Tlr7* and *Cysltr1*. We chose these X_m_-imprinted genes since they showed the most robust differences between X_m_ and X_p_ neurons and are also known to have human homologues, increasing potential human relevance. We first validated this technology in vitro using primary mouse neurons (Fig. 4a,b). Using lentiviral vectors expressing dCas9 under a neuronal promoter and single-guide RNAs (sgRNAs) (Extended Data Table 2) under a ubiquitous promoter to the three selected imprinted X genes, we co-transfected primary neurons and, nearly two weeks later, measured gene expression by RT–qPCR (Fig. 4a). We could not discern whether there was a CRISPRa-mediated increase from the X_m_ or X_p_ chromosomes using the current model. Since the three sgRNAs were positioned in the same construct, transfection simultaneously increased the expression of the three genes in a neuron when combined with dCas9. CRISPRa simultaneously increased the mRNA expression of *Sash3*, *Tlr7* and *Cysltr1* in neurons by approximately twofold (Fig. 4b).

## CRISPRa of X_m_ genes improves cognition

We then applied this validated CRISPRa approach in vivo in the hippocampus of old female mice (Fig. 4c,d). This approach achieved expression of both dCas9 and sgRNA lentiviral constructs in approximately 10% of dentate gyrus neurons, as determined by immunohistochemistry (Fig. 4c–e). Since a synapsin 1 promoter drove dCas9 expression, co-transfection targeted neuronal cell types. Although the stereotaxic injections were targeted to the hilus of the dentate gyrus, we cannot rule out the possibility of trans-synaptic or anterograde transport from transfected cells projecting to neurons in the CA1 and CA3 regions.

Through FACS sorting of old hippocampi transfected with either sgRNA with a scrambled sequence (control) or *Sash3*, *Tlr7* and *Cysltr1*, we next isolated neuronal nuclei and performed RNA-seq on this broader population of cells (Fig. 4f). Although a small fraction of these neurons underwent CRISPRa owing to co-transfection with dCas9, a heat map clearly showed an altered pattern of differentially expressed genes (DEGs) compared with controls (Fig. 4g). Pathway analysis to identify Gene Ontology terms that were most associated with DEGs predicted that upregulation of the silenced X genes stimulated pathways of mitochondrial energy production in the neuronal population at large, potentially improving cognitive functions in the ageing brain (Fig. 4h).

**Extended Data Fig. 8:**
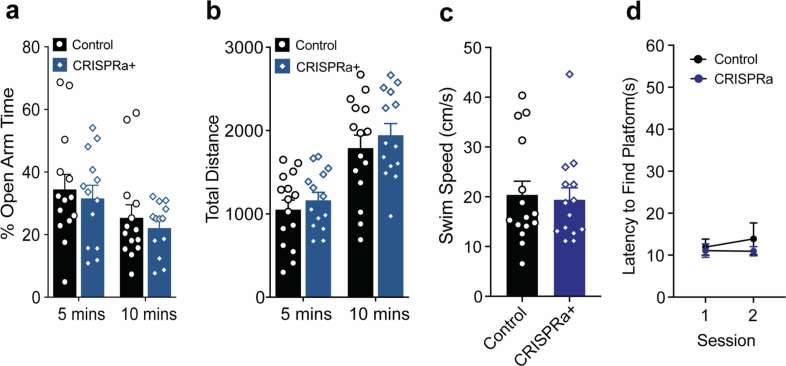
CRISPRa-mediated overexpression of Sash3, Tlr7 and Cysltr1 in old mice did not alter measures of anxiety in the EPM or swim speed in the MWM. **a**, Anxiety-like behavior, measured by % time in open arm, did not differ between groups at either 5 or 10 min (Control mice: n = 14; CRISPRa+ mice: n = 13; age = 19 months). **b**, Total distance traveled in the EPM, did not differ between groups at either 5 or 10 min (Control mice: n = 15; CRISPRa+ mice: n = 14; age = 19 months). **c**, Swim speed did not differ between the experimental groups, measured during the visible platform trials (Control mice: n = 15; CRISPRa+ mice: n = 14; age = 20 months), Unpaired two-tailed t-test, *P* > 0.05. **d**, Latency to find a visible platform did not differ between the experimental groups (Control mice: n = 15 (session 1), n = 14 (session 2); CRISPRa+ mice: n = 15 (session 1), n = 14 (session 2); age = 20 months). Two-way mixed model ANOVA: genotype *P* > 0.05. Data represent means ± SEM. Source Data

We tested whether a gain in function from increasing X_m_ imprinted genes, through CRISPRa-mediated upregulation of *Sash3*, *Tlr7* and *Cysltr1*, improved cognition in the ageing female brain. To this end, we performed a battery of behavioural and cognitive tests using old female mice transfected with dCas9 and either scrambled sgRNA or specific sgRNAs (Fig. 4i). In Morris water maze experiments, upregulation of X_m_-imprinted genes increased spatial learning (Fig. 4j) and memory of the platform location (Fig. 4k) compared with controls; percentage time spent in the target quadrant was measured since it showed a dynamic assay range for old mice. Swimming speeds and latency to find the visible platform did not differ between groups (Extended Data Fig. 8).

Likewise, in novel place recognition experiments, an independent and less stressful test of cognition, upregulation of X_m_-imprinted genes improved spatial learning and memory (Fig. 4l). In the EPM experiments, anxiety-like behaviour did not differ between the experimental groups (Extended Data Fig. 8), indicating that the gain of function was specific to spatial learning and memory. Collectively, these data show that CRISPRa of X_m_-imprinted genes functionally improved cognition in the ageing female brain.

## Discussion

We found that skew towards the X_m_ chromosome compared with X mosaicism increasingly impaired cognition across the lifespan of female mice. X_m_ and X_p_ were nearly identical in this group of studies, suggesting an epigenetic, parent-of-origin modulation of X-chromosome gene expression. In further studies of genetically identical X_m_ and X_p_ neurons from the same brain, X_m_ neurons accelerated brain ageing and underwent neuronal silencing of select genes, when directly compared with neighbouring X_p_ cells. Upregulation of selected X_m_-imprinted genes, *Sash3*, *Tlr7* and *Cysltr1*, in the hippocampus improved learning and memory and countered cognitive ageing in old female mice.

X_m_ skew impaired brain functions among multiple organ systems studied in healthy ageing female mice—and this impairment was increasingly observed across the lifespan. In the brain, more genes are expressed from the X chromosome than from any single autosome^36,37^; thus, the brain is probably more sensitive to alterations in X-chromosome-linked gene expression. In the context of X chromosomal abnormalities, female individuals with Turner’s syndrome (45,XO) experience greater cognitive impairment when the X chromosome is maternally compared with paternally inherited^17,38^, a finding also observed in mouse models of Turner’s syndrome^30,39^. However, even in the absence of mutations or X chromosomal abnormalities, our data suggest that the X_m_ chromosome, which is variably skewed in the population of typical female individuals with two X chromosomes, impairs neural functions.

We investigated X imprinting in a select population of hippocampal cells that express synapsin 1, a neuronal and synaptic marker^40^. Neurons were isolated through a density gradient followed by FACS sorting for fluorescence driven by synapsin 1. Assessing this subtype of hippocampal cells increases the relevance of X imprinting to mechanisms of learning and memory and represents an advance from previous assessments of whole brain or regional homogenates that necessarily combined multiple cell types. Our data in neuronal hippocampus cells indicate X_m_ silencing of cognition-related loci.

Most of the imprinted X-chromosomal genes in hippocampal neurons were of maternal origin. Notably, our data validate *Xlr3b* as a known paternally imprinted gene, as identified in previous studies^30,31^. X_p_-imprinted genes such as *Xlr3b* could also contribute to the impaired memory phenotype observed in X_m_ mice, as *Xlr3b* decreases dendritic spine numbers^41^, impairs dendritic transport and can lead to synaptic dysfunction^42^. Among the top maternally imprinted genes, *Tlr7* regulates genes crucial for memory and long-term potentiation^43,44^. The top maternally silenced genes *Sash3*, *Tlr7* and *Cysltr1* are involved in immune-related processes but their roles in neuronal functions have largely been unexplored. Their CRISPRa-mediated upregulation in the ageing brain predicted energetic modulation. These factors may be at the intersection between immune signalling, synaptic pruning of neurons by microglia^45^ and (perhaps relatedly) optimized energetics—all substrates of enhanced synaptic connectivity required for better cognition. The silencing of these X factors in X_m_ neurons, compared with their robust expression in X_p_ neurons, may thus impair substrates and signals of cognition. Notably, there could be a selective advantage to X_m_ imprinting, including of immune-related genes, early in the development of neurons to optimize synaptic pruning, development and health, an advantage lost after development.

Since male mice exclusively have an X_m_, they may show similarly increased cognitive ageing as observed in X_m_ female mice, a possibility that remains to be tested. However, they additionally have a Y chromosome, circulating androgens and lack escapee expression from a second X, which could also influence cognitive ageing and X_m_ imprinting. Understanding how X_m_ influences cognitive ageing and gene imprinting in XY males is important for future studies.

Upregulating X_m_ imprinted factors in a region in the hippocampus improved cognition in the ageing female brain. This suggests that X_m_-imprinting silences cognitive loci important in ageing. This is consistent with findings that manipulating key, albeit small, cell populations in functional hubs can influence larger neural networks^46,47^. CRISPRa-mediated, simultaneous increases in *Sash3*, *Tlr7* and *Cysltr1* in the dentate gyrus acutely enhanced cognition in the old brain, thereby countering cognitive ageing. Because the X_m_ chromosome imprints multiple genes, a broad approach to simultaneously upregulate the most robustly silenced genes enabled analysis of their collective function. Whether single X_m_-imprinted genes could mediate cognitive improvement in ageing remains to be investigated.

Our data suggest that female individuals with more skew towards an active X_m_ chromosome, even in the absence of mutations, could experience decreased cognitive functions—or could be at increased risk of neurodegenerative conditions such as Alzheimer’s disease—compared with those with more balanced X mosaicism in parent-of-X origin, particularly with age. This may be due to the absence of certain X genes in hippocampal neurons expressing the X_m_, a possibility that should be probed in human cell types. Understanding epigenetic parent-of-X silencing in neurons enables the unravelling of X-chromosome-derived pathways that can counter cognitive deficits and brain ageing.

## Methods

### Animals

The Institutional Animal Care and Use Committee of the University of California San Francisco (UCSF) approved all animal studies. Mice were kept on a 12 h light–dark cycle, with temperatures between 19 °C and 23 °C and humidity between 30% and 70% with ad libitum access to food and water. The standard housing conditions were five mice per cage except during Morris water maze experiments and metabolic tests for the Comprehensive Lab Animal Monitoring System (CLAMS), when mice were singly housed. All experiments were carried out during the light cycle with the exception of the CLAMS metabolic tests, for which data were collected throughout the light and dark cycles. To assess effects of X_m_ skew compared with mosaicism, we generated mice with global maternal X_m_-only expression (X_m_ mice) with non-transgenic littermates showing normal, random X inactivation (X_m_+X_p_ mice). X_m_-only expression was achieved by *Xist* deletion. Of note, *Xist* is a long noncoding RNA that regulates random X-chromosome inactivation. In brief, we crossed 129-Xist^tm2Jae^/Mmnc mice^10^ obtained from the Mutant Mouse Resource and Research Centers with *Zp3*^*cre*^ mice, provided by S. Kalantry^11^. F_2_ mice were then backcrossed to C57BL/6J mice to obtain a congenic C57BL/6J background, which was verified by genetic testing. Female mice underwent multiple tests of metabolism, cardiac function, body composition, behaviour and cognition during life stages indicated in the captions of Figs. 1–4. All arenas and equipment were cleaned with 70% ethanol between tests, except for the water maze. All experimenters were blind to mouse genotypes and groups.

To assess the differences between X_m_ and X_p_ neurons from the same brain, we used well-characterized mice obtained from the laboratory of J. Nathan^24^ that were generated to carry X-linked, Cre-activated and nuclear fluorescent reporters of GFP on one X chromosome and tdTomato on the other. They were also backcrossed to obtain a congenic C57BL/6J background. These reporter mice possess a floxed tdTomato fluorescent protein or a floxed GFP protein inserted into the *Hprt* locus of the X chromosome using modified *Hprt-*targeting vectors. The *Hprt* locus is subject to random X-chromosome inactivation and inserting the fluorescent proteins in this position ensures that once crossed with a suitable Cre line, either GFP or tdTomato is expressed from each cell but never both. The Cre line used to drive cell-type-specific X_m_ and X_p_ fluorescence was well characterized with a synapsin I, neuron-specific promoter^40^.

### Cardiac function

Measurements of cardiac function were performed as described^48^. In brief, mice were anaesthetized with isoflurane. Body temperature was monitored throughout the procedure using a rectal probe. A warm ultrasound gel was applied to the chest. Using a MX550S transducer, the B and M mode parasternal short-axis view was recorded, the diameter of the left ventricular lumen was measured and the ejection fraction was calculated. Afterwards, electrodes were removed, the ultrasound gel was removed and animals were allowed to recover before being returned to their cages.

### Body composition analysis

Body composition analysis was conducted as described^49,50^ with staff members at the metabolism core of the Nutrition and Obesity Research Center at the UCSF. The Lunar PIXImus densitometer (GE Medical Systems) was used to analyse body composition of each mouse using dual energy X-ray absorptiometry technology. In brief, mice were weighed and anaesthetized with avertin before being immobilized on a sticky mat. X-ray measurements were taken and the region of interest was adjusted to ensure the whole mouse was considered for the analysis. Data output provided bone, tissue and fat measurements.

### CLAMS metabolism

Metabolic analysis was conducted as previously described^49^ with staff members at the metabolism core of the Nutrition and Obesity Research Center at the UCSF. In brief, mice were singly housed for one week for habituation to the experimental conditions. Mice were then placed in the CLAMS and monitored for five days. Data were generated in 1 h bins and used to calculate metabolic parameters, including oxygen consumption (${V}_{{{\rm{O}}}_{2}}$), carbon dioxide production (${V}_{{{\rm{CO}}}_{2}}$), energy expenditure and respiratory exchange ratio.

### Morris water maze

Water maze testing was performed as described^51–54^. In brief, we filled the water maze pool (diameter, 122 cm) with white opaque water (21° ± 1 °C) and submerged a square 14 cm^2^ platform 2 cm below the surface. Mice underwent two pretraining trials that consisted of swimming through a channel to mount a rescue platform, before hidden training. The platform was kept in the same submerged spot during all hidden platform training trials; the location where mice were dropped into the pool varied between trials. For the hidden trials, mice received four trials daily for eight days. For the probe trials, the platform was removed, mice were allowed to swim for 60 s, and their latency to enter the previous platform area was recorded for young mice whereas the percentage of time spent in the target quadrant was recorded for old mice. In this study, the latency probe measure showed a dynamic range in young mice; by contrast, old mice showed a ceiling effect of the assay. In old mice, the percentage of time spent in the target quadrant was a sensitive probe measure as non-transgenic controls showed clear age-induced impairment whereas young mice showed a ceiling effect. Notably, probe measures and their sensitivities to memory in young and old mice can also vary between water maze studies. Following probe testing, mice were assessed for their ability to find the platform when it is marked with a visible cue (15 cm pole on the platform).

### Open field

Open-field testing was carried out as described^18^. In brief, mice were acclimatized to the room for 1 h before testing and allowed to explore the open field for 10 min. The open field consisted of a clear plastic chamber (41 × 30 cm) and total activity was detected by measuring beam breaks using an automated Flex-Field/Open Field Photobeam Activity System (San Diego Instruments). Visual cues included a fan, wires and a grid, and were located on three walls. For repeat testing, mice were placed in the same chamber. The index of forgetfulness was calculated by subtracting activity levels on the first day of testing in middle and old age from activity levels on the last day of testing of the previous life stage.

### EPM

EPM testing was carried out as described^51^. The room was maintained in dim light for both habituation and testing. In brief, mice were habituated to the testing room for 1 h before testing. Mice were placed in the centre of the EPM facing the open arm and allowed to explore for 10 min. Distance travelled and the percentage of time spent in the open versus closed arms was recorded using the Kinder Scientific Elevated Plus Maze and MotorMonitor system.

### Two-trial large Y maze

The two-trial large Y maze test (with visual cues at the end of each arm) was carried out as described^53,55^. Sixteen hours after a training session during which the novel arm was closed off, mice were returned to the two-trial large Y maze and allowed to explore all arms freely for 5 min. Time spent in the novel and familiar arm was recorded using the AnyMaze software and the novel to familiar ratio was calculated.

### Novel place recognition

Testing was carried out as described^56^. In brief, mice were acclimatized to the testing room for 1 h before testing, which was performed in a square white chamber (40 × 40 cm) under dim lighting. During the training session, mice were presented with two identical objects placed equidistant from each other and from the surrounding chamber walls. During this training session, mice showed a similar preference for each of the objects. For the test session 4 h later, one of the objects was moved to a new location and mice were allowed to explore for 10 min. Time of object exploration was obtained from the videos using the CleverSys TopScan Automated Behavior Analysis System (v.3.0) and analysed.

### Epigenetic DNA age analysis

Hippocampal tissue samples were flash-frozen. Samples then underwent sample library preparation and sequencing analysis as described (Zymo Research)^57^. In brief, genomic DNA was extracted using the Quick-DNA Miniprep plus kit and bisulfite converted using the EZ DNA Methylation Lightning kit. The samples were then enriched for sequencing of over 500 age-associated gene loci on an Illumina HiSeq 1500 instrument using 100-bp paired-end sequencing (X_m_ and X_m_+X_p_ hippocampal samples) or Illumina NovaSeq 6000 instrument using 150-bp paired-end sequencing (X_m_ and X_p_ hippocampal neurons; X_m_ and X_m_+X_p_ blood samples). Illumina’s base calling software was used to identify sequence reads and aligned to a reference genome using Bismark, an aligner optimized for bisulfite sequence calling (http://www.bioinformatics.babraham.ac.uk/projects/bismark/). The methylation level was determined by proportion of the numbers of ‘C’ reported to the total numbers of ‘C’ and ‘T’. Calculated DNA methylation values obtained from the sequence data were used to predict the epigenetic age using a proprietary DNAge predictor (Zymo).

### FACS

Fresh hippocampal tissue was first homogenized into single-cell suspension as previously described^58^. We enriched for neurons by applying the cell suspension to an Optiprep density gradient and collected only the neuronal fraction for FACS sorting^58^. Pre-enrichment for neurons through a density gradient was performed prior to FACS sorting of neurons for the young and old RNA-seq study, and not for the replicate RNA-seq study in young mice or the epigenetic analysis. For parent-of-X origin analysis, hippocampal cells were separated into X_m_ active and X_p_ active cells using a Sony SH800 FACS machine with a 100-mm cartridge at 40 psi. Cells were collected into a 15 ml flacon tube containing 2 ml sample collection buffer. Samples and collection tubes for sorting were kept at 4 °C during sorting. Hippocampi from six mice were pooled together to form a single sample. In total, five samples derived from 30 mice were prepared. Each sample was FACS-sorted into X_m_ cells (GFP^+^, green) and X_p_ cells (tdTomato^+^, red). For CRISPRa^+^ samples, we sorted dCas9^+^ and GFP^+^ nuclei using a 100 mm nozzle at 100 psi on a BD Biosciences FACSAria III. For both experiments, samples collected after sorting were centrifuged at 1,000 rpm for 10 min. The supernatant was discarded, and cells were resuspended in 250 ml of Trizol and stored at −80 °C until RNA-seq sample preparation and analysis.

### Neuronal nucleus isolation

Neuronal nuclei were isolated using the Nuclei EZ Prep Isolation kit (Sigma NUC-101). Frozen hippocampi were thawed on ice for 25 min before addition of 2 ml of ice-cold EZ lysis buffer. A hand-held homogenizer was used to completely homogenize the tissue while avoiding frothing. An additional 2 ml of ice-cold EZ lysis buffer was added and samples were then incubated on ice for 10 min. Samples were then centrifuged at 500*g* for 10 min at 4 °C. The supernatant was discarded and the pellet was resuspended in 1 ml ice-cold EZ lysis buffer. Once the pellet was properly resuspended, an additional 3 ml of ice-cold EZ lysis buffer was added and samples were incubated on ice for 15 min. Samples were then centrifuged at 500*g* for 10 min at 4 °C. The supernatant was discarded, and the pellet was resuspended in 350 ml of EZ storage buffer. Nuclei were filtered through a 30-mm cell strainer (MACS 130-041-407), counted and stored at −80 °C until FACS sorting.

### RNA-seq

In brief, RNA sequencing libraries were prepared using the SMART-Seq v4Ultra Low Input RNA Kit (Clontech). Paired-end reads were obtained using an Illumina HiSeq instrument. The quality of the reads was determined using FastQC, and more than 90% of reads from each sample had a mean quality score over 30. The trimmed reads were mapped to the *Mus musculus* GRCm38 reference genome available on ENSEMBL using the STAR aligner v.2.5.2b. Unique gene hit counts from exons were calculated using featureCounts in the Subread package v.1.5.2. Downstream differential expression analysis was performed using DESeq2. Sequencing was performed by Azenta Life Sciences.

### Identification of imprinted genes and RT–qPCR validation

Imprinted genes were selected on the basis of a list of criteria as follows: (1) significant *P* value and adjusted *P* value; (2) mean of normalized gene expression from one sample group of less than 50 and mean of normalized gene expression from the other sample group of over 100; (3) significant χ^2^ *P* value and adjusted significant χ^2^ *P* value; and (4) fold change above 10. On the basis of these criteria, we identified five imprinted genes. RT–qPCR was used to validate the expression of imprinted genes identified in the RNA-seq analysis. Primers were designed using the NCBI Primer Blast page and purchased from Integrated DNA Technologies. *Sash3* Fwd, CTGGCAGTGAAGAGGCTGAA, Rev, GACCCTGCAGTTGCTCTTCT; *Cysltr1* Fwd, GGTACCAGATAGAGGTCTCCC, Rev, CTCCAGGAATGTCTGCTTGGT; *Tlr13* Fwd, TCCTCCCTCCCTGGAGTTTT, Rev, AGGCACCTTCGTCGATCTTC; *Tlr7* Fwd, TGCACTCTTCGCAGCAACTA, Rev, ATGTCTCTTGCTGCCCCAAA; *Xlr3b* Fwd, AAAAGGAAGGCCACTGACAC, Rev, ACCAGCATCAAGGACTTCTCTG; *Gapdh* Fwd, GGGAAGCCCATCACCATCTT, Rev, GCCTTCTCCATGGTGGTGAA; 18S RNA Fwd, AGGGGAGAGCGGGTAAGAGA, Rev: GGACAGGACTAGGCGGAACA.

### Lentivirus production and stereotaxic injection

Simultaneous overexpression of *Sash3*, *Tlr7* and *Cysltr1* was achieved using a dCas9 Synergistic Activation Mediator Lentivirus (Lenti-hSyn-dCas9-VP64-p65-RTA-NLS-SV40-Puro virus) and a lentivirus containing sgRNAs for the three genes (Lenti-U6-Sash3 sgRNA-H1-Cysltr1 sgRNA-U6-Tlr7 sgRNA2-PGK-GFP). Two sets of sgRNAs were pooled together to improve efficiency of gene upregulation (Extended Data Table 2). A similar construct with a scrambled sequence (Lenti-U6-Scrambled sgRNA-PGK-GFP) was used as control (Extended Data Table 2). Active lentiviral particles were obtained from Applied Biological Materials (ABM). ABM performed lentiviral packaging in HEK293T cells using a second-generation co-transfection system. HEK293T cells were subcultured at 70% density in a 15-cm dish one day before virus production. The following day, transfection was performed using 180 μg total plasmid DNA (60 μg expression vector, 120 μg second-generation packaging mix (ABM LV003) and 80 µl lentifectin (ABM G2500)) in the absence of serum for 5 h before restoring the culture conditions back to DMEM + 5% FBS. Then 72 h after transfection, supernatant viruses were collected, purified and stored in PBS storage buffer. The final recombinant lentivirus were validated by titre and HEK293T transduction to ensure the virus were free from bacteria and mycoplasma contamination. Next 18-month-old C57BL6 wild-type mice were anaesthetized using isoflurane at 2–3% and placed in a stereotaxic frame. Then 5 µl of lentiviral vectors (2.5 μl dCas9 + 2.5 µl sgRNA per hemisphere) were stereotactically injected bilaterally into the dentate gyrus of the hippocampus using the coordinates, anteroposterior = −2.1, mediolateral = ±1.7 and dorsoventral = 1.9. After surgery, mice were allowed to wake up completely on a heating pad before being returned to their home cage. All behavioural assays were conducted beginning at 4 weeks and ending at 16 weeks after lentiviral injections.

### Immunofluorescence microscopy

Immunofluorescence was performed as previously described^59^. In brief, mice were perfused with cold PBS (10 ml min^−1^) for 5 min using a peristaltic pump. Whole brains were then collected and post-fixed in 4% paraformaldehyde for 48 h and subsequently preserved in 30% sucrose (prepared in PBS). Whole brains were sectioned coronally at 40 μm thickness on a freezing sliding microtome throughout the entire hippocampus. Sections were stored in the cryoprotective medium at −20 °C. Free-floating sections were blocked with donkey serum and incubated with primary antibodies at 4 °C overnight at the following concentration for microscopy: rabbit anti-GFP (1:1,000, Sigma G1544) and mouse anti-dCas9 (1:500, Invitrogen MA523519). After washing, sections were incubated with donkey anti-rabbit Alexa Fluor 488 (1:1,000, Thermo Fisher, A32790) and donkey anti-mouse Alexa Fluor 594 (1:1,000, Thermo Fisher, A21203) at room temperature for 2 h. DAPI (300 nM) was added during the last 10 min of the 2 h incubation at room temperature. Sections were washed and mounted with Vectashield before imaging on digital fluorescence microscope with spinning-disk confocal system (Nikon CSU-W1).

### Statistical analysis

Experimenters were blinded to genotype. Statistical analyses were carried out using GraphPad Prism (v.7.0) for *t*-tests and two-way ANOVAs and R Studio (v.2.0) for mixed-model ANOVAs and post-hoc tests. All tests were two tailed unless indicated otherwise. Differences between two means were assessed using unpaired *t*-tests and a two-way ANOVA to assess differences among multiple means for all experiments unless otherwise stated. Post-hoc tests were conducted with Bonferroni–Holm correction in R to control for a family-wise error rate at *α* = 0.05 when rounded to two decimal points, unless indicated otherwise. A mixed-model ANOVA was used to analyse Morris water maze and open-field data and included effects for repeated measures. Exclusion criteria (greater than 2 s.d. above or below the mean) were defined a priori to ensure unbiased exclusion of outliers in mouse behaviour studies. Error bars represent the s.e.m. and null hypotheses were rejected at or below a *P* value of 0.05 when rounded to two decimal points. Linear models were fitted in R using the standard lme package.

### Reporting summary

Further information on research design is available in the Nature Portfolio Reporting Summary linked to this article.

## Online content

Any methods, additional references, Nature Portfolio reporting summaries, source data, extended data, supplementary information, acknowledgements, peer review information; details of author contributions and competing interests; and statements of data and code availability are available at 10.1038/s41586-024-08457-y.

## Supplementary information

Reporting Summary

## Source data

Source Data Fig. 1

Source Data Fig. 2

Source Data Fig. 3

Source Data Fig. 4

Source Data Extended Data Fig. 2

Source Data Extended Data Fig. 3

Source Data Extended Data Fig. 4

Source Data Extended Data Fig. 6

Source Data Extended Data Fig. 7

Source Data Extended Data Fig. 8

## Figures and Tables

**Extended Data Table 1:**
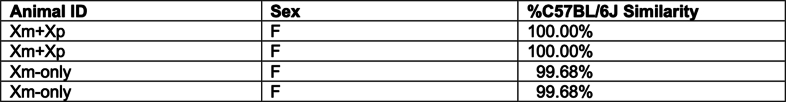
Mouse Genome Scan SNP analysis showing Xm-only mice are 99.68% C57BL/6 J due to minor heterozygosity on the X chromosome

**Extended Data Table 2:**
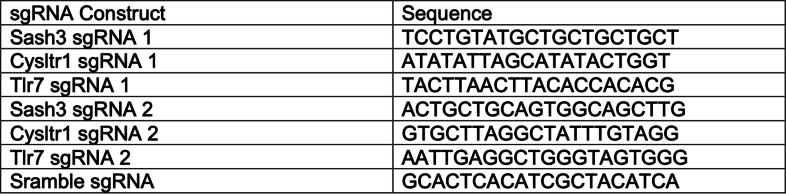
sgRNA sequences for CRISPR activation-mediated overexpression of Tlr7, Sash3 and Cysltr1

## Data Availability

Transcriptomic data for samples discussed in this publication have been deposited in the NCBI Gene Expression Omnibus and are accessible through GEO Series accession number GSE200461 (X_m_ versus X_p_ samples) and GSE280893 (control versus CRISPRa^+^ samples). Source data are provided with this paper.
