# Supplementary material for: The maternal X chromosome affects cognition and brain ageing in female mice
Source: Nature. 2025 Jan 22;638(8049):152–9. doi: 10.1038/s41586-024-08457-y (PMC11798838; doi:10.1038/s41586-024-08457-y)
Supplement: Supplementary file 1 — Reporting Summary [file 41586_2024_8457_MOESM1_ESM.pdf]

nature portfolio

Corresponding author(s): Dena Dubal

Last updated by author(s): Nov 8, 2024

Reporting Summary

Nature Portfolio wishes to improve the reproducibility of the work that we publish. This form provides structure for consistency and transparency in reporting. For further information on Nature Portfolio policies, see our Editorial Policies and the Editorial Policy Checklist.

Statistics

For all statistical analyses, confirm that the following items are present in the figure legend, table legend, main text, or Methods section.

n/a	Confirmed	
☐	☒	The exact sample size (*n*) for each experimental group/condition, given as a discrete number and unit of measurement
☐	☒	A statement on whether measurements were taken from distinct samples or whether the same sample was measured repeatedly
☐	☒	The statistical test(s) used AND whether they are one- or two-sided<br>*Only common tests should be described solely by name; describe more complex techniques in the Methods section.*
☒	☐	A description of all covariates tested
☐	☒	A description of any assumptions or corrections, such as tests of normality and adjustment for multiple comparisons
☐	☒	A full description of the statistical parameters including central tendency (e.g. means) or other basic estimates (e.g. regression coefficient) AND variation (e.g. standard deviation) or associated estimates of uncertainty (e.g. confidence intervals)
☐	☒	For null hypothesis testing, the test statistic (e.g. *F*, *t*, *r*) with confidence intervals, effect sizes, degrees of freedom and *P* value noted<br>*Give P values as exact values whenever suitable.*
☒	☐	For Bayesian analysis, information on the choice of priors and Markov chain Monte Carlo settings
☒	☐	For hierarchical and complex designs, identification of the appropriate level for tests and full reporting of outcomes
☒	☐	Estimates of effect sizes (e.g. Cohen's *d*, Pearson's *r*), indicating how they were calculated

Our web collection on statistics for biologists contains articles on many of the points above.

Software and code

Policy information about availability of computer code

Data collection	MX550S transducer, Lunar PIXImus Densitometer, Comprehensive Lab Monitoring System, Flex-Field/Open Field Photobeam Activity System, Kinder Scientific Elevated Plus Maze and MotorMonitor System, Anymaze Software (version 6.35), Noldus Ethovision (version 10, Noldus), Illumina HiSeq, Sony SH800 Cell Sorter, BD Biosciences FACSAria III Cell Sorter.
Data analysis	FastQC, STAR aligner v.2.5.2b, Subread package v.1.5.2, DESeq, Graphpad prism (version 7.0), R-studio (v 4.2), TopScan Automated Behavior Analysis System (CleverSys, v3.0)

For manuscripts utilizing custom algorithms or software that are central to the research but not yet described in published literature, software must be made available to editors and reviewers. We strongly encourage code deposition in a community repository (e.g. GitHub). See the Nature Portfolio guidelines for submitting code & software for further information.

Data

Policy information about availability of data

All manuscripts must include a data availability statement. This statement should provide the following information, where applicable:

- Accession codes, unique identifiers, or web links for publicly available datasets
- A description of any restrictions on data availability
- For clinical datasets or third party data, please ensure that the statement adheres to our policy

Transcriptomic data for samples discussed in this publication have been deposited in NCBI's Gene Expression Omnibus and are accessible through GEO Series accession number GSE200461 (Xm v Xp samples) and GSE280893 (Control v CRISPRa+ samples).

Research involving human participants, their data, or biological material

Policy information about studies with human participants or human data. See also policy information about sex, gender (identity/presentation), and sexual orientation and race, ethnicity and racism.

Reporting on sex and gender	N/A
Reporting on race, ethnicity, or other socially relevant groupings	N/A
Population characteristics	N/A
Recruitment	N/A
Ethics oversight	N/A

Note that full information on the approval of the study protocol must also be provided in the manuscript.

Field-specific reporting

Please select the one below that is the best fit for your research. If you are not sure, read the appropriate sections before making your selection.

☒ Life sciences ☐ Behavioural & social sciences ☐ Ecological, evolutionary & environmental sciences

For a reference copy of the document with all sections, see nature.com/documents/nr-reporting-summary-flat.pdf

Life sciences study design

All studies must disclose on these points even when the disclosure is negative.

Sample size	A variance and effect size for behavioral studies were previously determined from pilot data and used to estimate the sample size necessary to detect a true difference 80% of the time at the alpha=0.05 significance level. Based on our experience and power calculations, typical group sizes for mouse experiments have been n=10-18 mice for molecular and behavioral studies.
Data exclusions	In mouse behavior and physiology studies, exclusion criteria (greater than 2 SDs above or below the mean) were defined a priori to ensure unbiased exclusion of outliers.
Replication	As part of our due diligence and commitment to the highest standards for reproducible research, we blind experimenters to genotype and randomize animals to experimental groups. Significant results are replicated when possible such as in behavior studies. Significant behavior experiments were replicated in at least one other independent test. RNA sequencing findings were replicated using qPCR.
Randomization	Groups for behavioral and physiology experiments were randomized.<br>For RNA-seq, samples were randomly assigned to a group based on the parent-of-orgin of the active X as determined by FACS analysis.
Blinding	We blind experimenters to genotype for all studies.

Reporting for specific materials, systems and methods

We require information from authors about some types of materials, experimental systems and methods used in many studies. Here, indicate whether each material, system or method listed is relevant to your study. If you are not sure if a list item applies to your research, read the appropriate section before selecting a response.

Materials & experimental systems

n/a	Involved in the study	
☐	☒	Antibodies
☐	☒	Eukaryotic cell lines
☒	☐	Palaeontology and archaeology
☐	☒	Animals and other organisms
☒	☐	Clinical data
☒	☐	Dual use research of concern
☒	☐	Plants

Methods

n/a	Involved in the study	
☒	☐	ChIP-seq
☐	☒	Flow cytometry
☒	☐	MRI-based neuroimaging

Antibodies

Antibodies used	Rabbit anti-GFP (1:1000, Sigma G1544) , mouse anti-dCas9 (1:500, Invitrogen MA523519), donkey anti-rabbit Alexa Fluor 488 (1:1000, Thermo Fisher, A32790), donkey anti-mouse Alexa Fluor 594 (1:1000, Thermo Fisher, A21203), 300nM of 4′,6-diamidino-2-phenylindole (DAPI).
Validation	Rabbit anti-GFP (1:1000, Sigma G1544) has been validated by Sigma in immunohistochemistry. This antibody has also been validated for immunohistochemistry in other studies (Liu et al, 2017, Development) where the antibody was used to stain for GFP+ transgenic zebrafish embryos. Mouse anti-dCas9 was validated for immunohistochemistry by Invitrogen. This antibody has also been validated for immunohistochemistry in other studies (Levy et al, 2020, Nat Biomed Eng) where the antibody was used to stain for Cas9 expression during CRISPR-mediated editing of the mouse brain.

Eukaryotic cell lines

Policy information about cell lines and Sex and Gender in Research

Cell line source(s)	Applied Biological Materials use early passage HEK 293T cells they have in house
Authentication	Cells were authenticated to be HEK 293T cells by Applied Biological materials
Mycoplasma contamination	No mycoplasma contamination was detected
Commonly misidentified lines (See ICLAC register)	NA

Animals and other research organisms

Policy information about studies involving animals; ARRIVE guidelines recommended for reporting animal research, and Sex and Gender in Research

Laboratory animals	This study used young (4-8 months), middle-aged (9-17 months) and old (18-24 months) maternal X only expressing transgenic mouse (Mus musculus) models on a C57BL/6J background. Young Zp3-Cre mice (3-7 months) were mated to 129-Xisttm2Jae/Mmnc mice to generate Xm-only mice. Syn-Cre was used to drive GFP and tdTomato expression specifically in neurons in both young (4-8 months) and old (18-24 months) mice. .
Wild animals	This study did not involve wild animals.
Reporting on sex	All studies used female mice to focus on the effects of X chromosomes without the confounds of the Y chromosome and androgens.
Field-collected samples	This study did not include samples collected from the field.
Ethics oversight	All studies were approved by the Institutional Animal Care and Use Committee of the University of California, San Francisco, and conducted in compliance with NIH guidelines.

Note that full information on the approval of the study protocol must also be provided in the manuscript.

Plants

Seed stocks	N/A
Novel plant genotypes	N/A
Authentication	N/A

Flow Cytometry

Plots

Confirm that:

☒ The axis labels state the marker and fluorochrome used (e.g. CD4-FITC).

☒ The axis scales are clearly visible. Include numbers along axes only for bottom left plot of group (a 'group' is an analysis of identical markers).

☒ All plots are contour plots with outliers or pseudocolor plots.

☒ A numerical value for number of cells or percentage (with statistics) is provided.

Methodology

Sample preparation	Briefly, mouse brains were collected into Hibernate A with B27 and glutamate (HABG) at 4 degrees celsius. The hippocampus was then dissected, diced and and transferred first into 2 ml Hibernate A (HA) at room temperature for 2 minutes, and then into 5 ml papain solution with Hoechst dye at 30 degrees for 30 minutes while shaking at approximately 170 rpm. Tissue pieces were transferred into warm (37 degrees celsius) HABG and mechanically triturated 10 times using stainless steel needles with decreasing inner diameter. In between trituration steps, remaining pieces were allowed to settle for 2 minutes, the top 1 ml of solution was transferred through a 45 uM cell strainer to a new tube and replaced with fresh HABG medium for nest trituration with the next smaller needle. After, the cell suspension was centrifuged at 800 g in a swinging bucket centrifuge for 15 minutes at 22 degrees celsius. The supernatant was discarded and cells were resuspended in 4ml of media. Cells were enriched for neurons by applying the cell suspension to an Optiprep density gradient. The neuronal fraction was collected and diluted with 4ml of media. The suspension was centrifuged in a swinging bucket centrifuge for 5 minutes at 22 degrees celsius. After, the supernatant was discarded and cells were resuspended in 500 ul of HABG without phenol red and stored on ice until sorted.
Instrument	Sony SH800 Cell Sorter.
Software	SONY SH800 Cell Sorter Software.
Cell population abundance	Percentage of GFP+ cells (Xm) and tdT+ cells (Xp) cells varied with each sample based on X chromosome inactivation patterns of the hippocampus of each mouse.
Gating strategy	Extended Figure 5 contains our FACS gating strategy. Cells were sorted by FSC and SSC to obtain single cells, then sorted for GFP+ and tdT+ cells.

☒ Tick this box to confirm that a figure exemplifying the gating strategy is provided in the Supplementary Information.
